# The Landmark Series: Minimally Invasive Pancreatic Resection

**DOI:** 10.1245/s10434-020-09335-3

**Published:** 2020-12-19

**Authors:** Jony van Hilst, Nine de Graaf, Mohammad Abu Hilal, Marc G. Besselink

**Affiliations:** 1 Department of Surgery, Cancer Center Amsterdam, Amsterdam UMC, Amsterdam, The Netherlands; 2 Department of Surgery, OLVG, Amsterdam, The Netherlands; 3 Department of Surgery, Instituto Ospedaliero Fondazione Poliambulanza, Brescia, Italy

## Abstract

**Background:**

Pancreatic resections are among the most technically demanding procedures, including a high risk of potentially life-threatening complications and outcomes strongly correlated to hospital volume and individual surgeon experience. Minimally invasive pancreatic resections (MIPRs) have become a part of standard surgical practice worldwide over the last decade; however, in comparison with other surgical procedures, the implementation of minimally invasive approaches into clinical practice has been rather slow.

**Objective:**

The aim of this study was to highlight and summarize the available randomized controlled trials (RCTs) evaluating the role of minimally invasive approaches in pancreatic surgery.

**Methods:**

A WHO trial registry and Pubmed database literature search was performed to identify all RCTs comparing MIPRs (robot-assisted and/or laparoscopic distal pancreatectomy [DP] or pancreatoduodenectomy [PD]) with open pancreatic resections (OPRs).

**Results:**

Overall, five RCTs on MIPR versus OPR have been published and seven RCTs are currently recruiting. For DP, the results of two RCTs were in favor of minimally invasive distal pancreatectomy (MIDP) in terms of shorter hospital stay and less intraoperative blood loss, with comparable morbidity and mortality. Regarding PD, two RCTs showed similar advantages for MIPD. However, concerns were raised after the early termination of the third multicenter RCT on MIPD versus open PD due to higher complication-related mortality in the laparoscopic group and no clear other demonstrable advantages. No RCTs on robot-assisted pancreatic procedures are available as yet.

**Conclusion:**

At the current level of evidence, MIDP is thought to be safe and feasible, although oncological safety should be further evaluated. Based on the results of the RCTs conducted for PD, MIPD cannot be proclaimed as the superior alternative to open PD, although promising outcomes have been demonstrated by experienced centers. Future studies should provide answers to the role of robotic approaches in pancreatic surgery and aim to identity the subgroups of patients or indications with the greatest benefit of MIPRs.

Minimally invasive pancreatic resections (MIPRs) have become a part of standard surgical practice worldwide, since their introduction in the early 1990s.^1^,^2^ This has resulted in numerous case-series and registry studies on minimally invasive distal pancreatectomy (MIDP) and minimally invasive pancreatoduodenectomy (MIPD).^3^–^11^ These studies reported less intraoperative blood loss, lower morbidity, and shorter hospital stay after MIPR (compared with the conventional open approach). However, there were also some concerns caused by high conversion rates, inferior oncological outcomes, and increased mortality reported in low-volume centers; these concerns hampered further widespread introduction of MIDP and MIPD.

To ensure the safe introduction of MIPRs, several training programs for both procedures were developed.^12^–^15^ These training programs included video and virtual reality training, biotissue drills, and on- and off-site proctoring. The LAELAPS-1 training program for MIDP in The Netherlands resulted in a sevenfold increase in the use of this technique, lower conversion rates (from 38 to 8%), less blood loss, and shorter hospital stay compared with the outcomes before training.^12^,^16^

Compared with distal pancreatectomy, the introduction of MIPD has been relatively slow, probably caused by the complexity of the procedure and the questionable benefits compared with the open approach. Therefore, specific, more extended, training programs were also developed for robot-assisted and laparoscopic pancreatoduodenectomy enhancing the introduction of these procedures.^13^,^17^

According to the IDEAL framework (Idea, Development, Exploration, Assessment, and Long-term study) for surgical innovation, all new surgical interventions should preferably be assessed in a randomized controlled trial (RCT).^18^ In recent years, several RCTs on MIPR have been developed and completed. In the present paper, we would like to provide an overview of all published and ongoing RCTs on MIPR.

## Methods

In order to provide an overview of all ongoing and published interventional studies on MIPR, we performed a search of the World Health Organization (WHO) trial registry database (September 2020). This database combines information from the largest international and national clinical trial databases. The following keywords were used: pancreas, minimally invasive, laparoscopy, and robot. The search was checked by two individual researchers and relevant trials were identified. Randomized trials were eligible when they compared MIPR (robot-assisted and/or laparoscopic distal pancreatectomy or pancreatoduodenectomy) using an open approach with these procedures. Allocation of patients had to be performed by randomization. All identified RCTs were checked and cross-checked on Pubmed for published study protocol or final trial report.

## Results

### Identified Randomized Controlled Trials

**Fig. 1:**
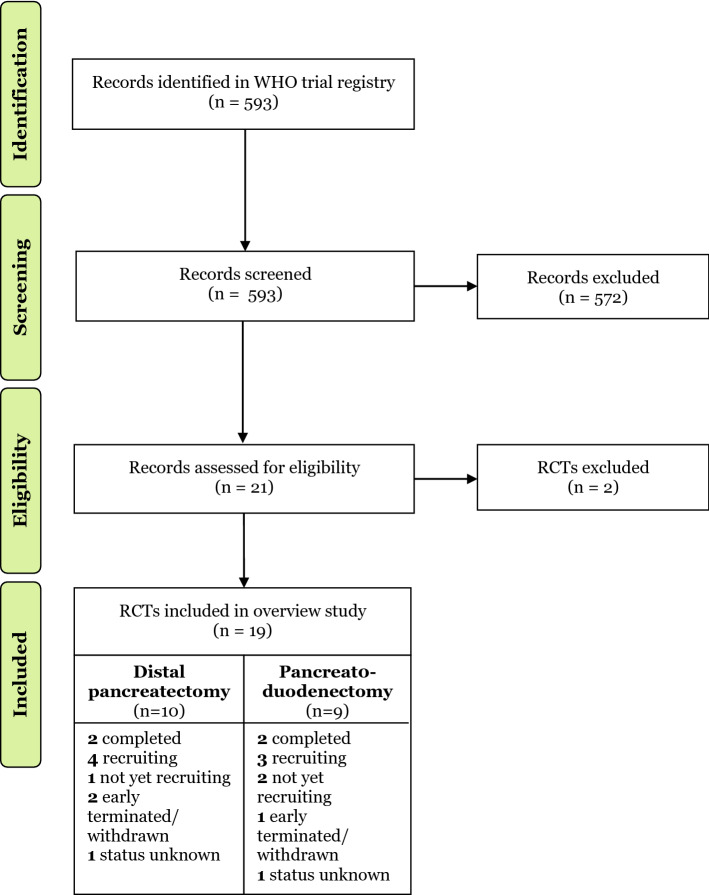
Included randomized controlled trials, *WHO* World Health Organization, *RCTs* randomized controlled trials

**Fig. 2:**
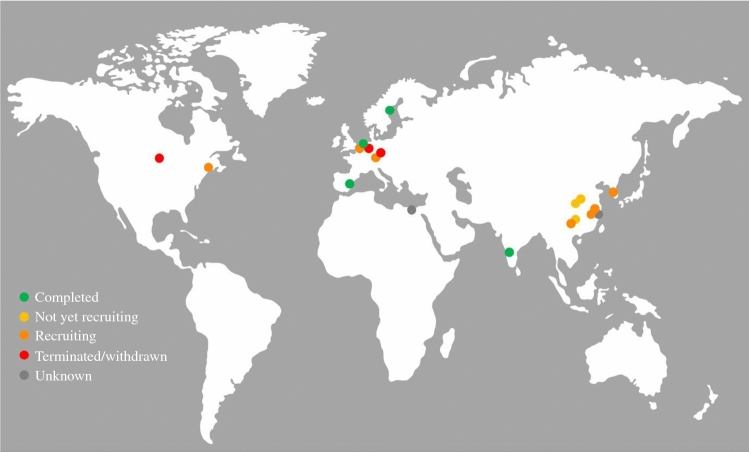
Worldwide spread of performed randomized controlled trials

The search of the WHO trial registry identified 95 RCTs, of which 19 were eligible (Fig. 1), from 11 different countries (Fig. 2).

### Minimally Invasive Distal Pancreatectomy

The search identified 10 RCTs on MIDP (Table 1). Two trials were terminated early due to financial reasons. One trial from the US compared laparoscopic distal pancreatectomy (LDP) with open distal pancreatectomy (ODP; NCT00988793). The German DAVID trial (NCT02269683) was designed to compare robot-assisted distal pancreatectomy (RDP) with LDP. Two trials from China have an unknown status. One trial was designed to compare circulating tumor cells after open, ‘no-touch’, and laparoscopic distal pancreatectomy for pancreatic cancer (NCT02451384). The other trial (NCT03770559) compared laparoscopic and open radical antegrade modular pancreatosplenectomy in patients with pancreatic cancer. This trial was registered in 2018 but is still not recruiting. The published and currently ongoing trials are discussed below.

**Table 1:** Randomized controlled trials on minimally invasive distal pancreatectomy

Trial	Country	Publication year	Funding	Arms	Primary outcome	Design	*N*	Outcomes	Status
LEOPARD^16^ NTR5188	Netherlands	2019	Private (J&J Med)	MIDP vs. ODP	TTFR	Multicenter Patient-blinded	51 MIDP (5 RDP/46 LDP) vs. 57 ODP	MIDP: ↓ TTFR ↓ LOHS ↓ DGE = complications = costs ↑ quality of life	Completed
LAPOP^19^,^31^ ISRCTN26912858	Sweden	2020	Public	LDP vs. ODP	LOHS	Single-center Non-blinded	60 LDP vs. 58 ODP	LDP: ↓ LOHS ↓ TTFR ↓ blood loss	Completed
DIPLOMA NCT04483726	International	Expected 2021	Private (J&J Med and Medtronic)	MIDP vs. ODP	R0 margin	Multicenter Patient- and assessor-blinded	258 sample size	NA	Recruiting
NCT03792932	China	Expected 2024	Public	LDP vs. ODP	2-year disease-free survival	Multicenter Non-blinded	306 sample size	NA	Recruiting
NCT03957135	Korea	Expected 2025	Public	LDP vs. ODP	2-year survival	Multicenter Non-blinded	244 sample size	NA	Recruiting
DISPACT-2 DRKS00014011	Germany	NA	Public	MIDP (RDP and LDP) vs. ODP	CCI	Single-center	294 sample size	NA	Recruiting
DAVID NCT02269683	Germany	NA	NA	RDP vs. LDP	R1 resection rate	Single-center Patient- and assessor-blinded	–	NA	Withdrawn/financial
NCT00988793	USA	NA	NA	LDP vs. ODP	Blood loss, overall complication rate, length of hospital stay	Single-center Non-blinded	128 sample size	NA	Terminated/financial
NCT03770559	China	Expected 2020	NA	LDP-RAMPS vs. ODP RAMPS	Overall survival	Single-center Non-blinded	60 sample size	NA	Not yet recruiting
NCT02451384	China	NA	NA	ODP vs. ‘no-touch’ vs. LDP	Circulating tumor cell count	Single-center Non-blinded	45 sample size	NA	Unknown

*MIDP* minimally invasive distal pancreatectomy, *ODP* open distal pancreatectomy, *LDP* laparoscopic distal pancreatectomy, *RDP* robot-assisted distal pancreatectomy, *RAMPS* radical antegrade modular pancreatosplenectomy, *TTFR* time to functional recovery, *LOHS* length of hospital stay, *DGE* delayed gastric emptying, *CCI* Comprehensive Complication Index, *NA* not available, ↓ indicates reduction, ↑ indicates increase, = indicates comparable outcome

#### LEOPARD

LEOPARD is a multicenter, patient-blinded, RCT that was performed in The Netherlands.^16^ Adult patients with left-sided pancreatic tumors (benign and malignant) without vascular involvement were included and randomized in a 1:1 ratio to MIDP (laparoscopic and robot-assisted) or ODP. Primary outcome was the time to functional recovery, which was defined as independently mobile at the preoperative level, pain control with oral medication alone, ability to maintain at least 50% daily required caloric intake, no intravenous fluid administration, and no clinical signs of infection when other criteria were met.

LEOPARD included 108 patients from 14 centers between April 2015 and March 2017, of whom 51 were randomized to MIDP and 57 to ODP. Time to functional recovery was 4 days (interquartile range [IQR] 3–6) after MIDP vs. 6 days (IQR 5–8) after ODP (*p* < 0.001). The conversion rate of MIDP to ODP was 8%. Operative blood loss was less after MIDP (150 vs. 400 mL; *p* < 0.001). However, operative time was longer after MIDP (217 vs. 179 min; *p* = 0.005), and delayed gastric emptying grade B/C was lower after MIDP (6% vs. 20%; *p* = 0.04). The Clavien–Dindo grade III or higher complication rate, postoperative pancreatic fistulas grade B/C, and 90-day mortality did not differ significantly between MIDP and ODP. Quality of life (days 3–30) was better after MIDP compared with ODP, and overall costs were non-significantly less after MIDP.

Additional analyses were performed for costs and quality of life up to 1 year after surgery. Total medical costs were comparable after MIDP (considering the low amount of robot-assisted procedures, included costs for this type of surgery were discarded) and ODP {mean difference −€911 (95% bias-corrected and accelerated confidence interval [CI] −€5190 to 3105); *p* = 0.680}. MIDP was shown to have a probability of at least 0.653 of being more cost effective (willingness-to-pay threshold of €0 per day of earlier recovery) compared with ODP, and 0.698 when society is willing to pay €80,000 per additional QALY. No significant differences in median cosmetic satisfaction scores and disease-specific QOL were seen.

#### LAPOP

The LAPOP trial is a Swedish, unblinded, parallel-group, single-center, superiority trial.^19^ Inclusion criteria were comparable with the LEOPARD trial (adult patients with a tumor confined to the pancreas). Patients were randomized in a 1:1 ratio to LDP or ODP. Primary outcome was postoperative hospital stay. In total, 58 patients were included—29 in the LDP group and 29 in the ODP group. The median postoperative hospital stay was 5 days (IQR 4–5) after LDP versus 6 days (IQR 5–7) in the open group (*p* = 0.002), and time to functional recovery was a median of 4 days (IQR 2–6) versus 6 days (IQR 4–7), respectively (*p* = 0.007). Operative time was comparable, i.e. 120 min for both groups (*p* = 0.482), and blood loss was less in the LDP group (50 vs. 100 mL; *p* = 0.018). Clavien–Dindo grade III or higher complications, grade B/C delayed gastric emptying, and grade B/C postoperative pancreatic fistulas were comparable for both groups.

#### Ongoing Trials

The search identified four ongoing trials on MIDP.

First, the DIPLOMA trial, an international RCT comparing MIDP (laparoscopic and robot-assisted) and ODP for patients with a PDAC in 30 centers from 11 countries (trial registry: NCT04483726). The primary outcome of DIPLOMA is microscopical radical (R0) resection margins, and the most important secondary outcome is survival. Inclusion of the required 258 patients started in 2019 is reportedly running on schedule. Outcomes are expected by the end of 2021.

Second, a multicenter prospective, non-inferiority, non-blinded, RCT that started recruiting in Korea in 2019 (trial registry: NCT03957135). This trial is also focusing on oncological outcomes and will include 244 patients with PDAC of the pancreatic body and tail, without evidence of distant metastasis or direct invasion of adjacent organs. Patients will be randomly allocated to either LDP or ODP and the primary endpoint of this trial is 2-year survival. Results of this trial are expected in 2025.

Third, a multicenter prospective, non-blinded RCT from China that has been recruiting since the beginning of 2019 (trial registry: NCT03792932). An estimated number of 306 patients with malignant pancreatic tumors (not further defined) of the body or tail will be randomized to either LDP or ODP. The primary endpoint of this trial is 2-year disease-free survival. Results of this trial are expected to be published in 2024.

Fourth, the DISPACT-2 trial (trial registry: DRKS00014011), a German, single-center, patient- and assessor-blinded RCT on MIDP versus ODP that has been recruiting since 2020. An estimated number of 294 patients with benign, premalignant or malignant indication for distal pancreatectomy will be randomized to either MIDP or ODP. The primary endpoint of this trial is postoperative mortality and morbidity assessed with the Comprehensive Complication Index (CCI) 3 months after intervention.

### Minimally Invasive Pancreatoduodenectomy

The search identified nine trials on MIPD (Table 2). Three trials are not yet recruiting or have an unknown status. One trial from Egypt (NCT02807701) was registered in 2016 and was designed to compare LPD with OPD for the duration of the hospital stay but currently has an unknown status. Two trials were registered in 2018 but are still not recruiting: the LOPA trial (NCT03747588), comparing LPD with OPD for patients with pancreatic cancer, and the TJDBPS07-trial (NCT03785743), designed in China to compare LPD with OPD in terms of overall survival. Three RCTs have been published and three are currently recruiting; these trials will be discussed below.

**Table 2:** Randomized controlled trials on minimally invasive pancreatoduodenectomy

Study site	Country	Year	Funding	Arms	Primary outcome	Design	*N*	Outcomes	Status
PLOT^20^ NCT02081131	India	2017	Public	LPD vs. OPD	LOHS	Single-center Non-blinded	32 LPD vs. 32 OPD	LPD: ↓ LOHS = complications = R0 resection rate Conversion rate 3%	Completed
PADULAP^21^ ISRCTN93168 938	Spain	2018	Public	LPD vs. OPD	LOHS	Single-center Non-blinded	34 LPD vs. 32 OPD	LPD: ↓ LOHS = complications = R0 resection rate = lymph node retrieval Conversion rate 23.5%	Completed
LEOPARD-2^22^ NTR5689	Netherlands	2019	Public and private (J&J Med)	LPD vs. OPD	Morbidity and mortality (phase II) TTFR (phase III)	Phase II/III Multicenter Patient-blinded	50 LPD vs. 49 OPD	LPD: ↑ TTFR and LOHS ↑ 90-day complication- related mortality = CD grade III or higher = grade B/C POPF Conversion rate 20%	Early terminated, published
TJDBPS01^23^ NCT03138213	China	Expected 2022	Public	LPD vs. OPD	LOHS	Multicenter Triple-blinded	656 sample size	NA	Recruiting
PORTAL NCT04400357	China	Expected 2024	Private (Intuitive)	RPD vs. OPD	TTFR	Phase III Multicenter Patient-blinded	244 sample size	NA	Recruiting
NCT04171440	USA	Expected 2025	Public	MIPD (RPD or LPD) vs. OPD	TTFR	Phase III Single-center Patient-blinded	240 sample size	NA	Recruiting
LOPA trial NCT03747588	China	NA	NA	LPD vs. OPD	Overall complications, POPF, intra-abdominal bleeding and intra-abdominal infection	Single-center Non-blinded	100 sample size	NA	Not yet recruiting
TJDBPS07 trial NCT03785743	China	Expected 2026	Public	LPD vs. OPD	5-year overall survival, disease-free survival	Multicenter Assessor-blinded	200 sample size	NA	Not yet recruiting
NCT02807701	Egypt	NA	Public	LPD vs. OPD	LOHS	Single-center Patient- and assessor-blinded	40 sample size	NA	Unknown

*MIPD* minimally invasive pancreatoduodenectomy, *OPD* open pancreatoduodenectomy, *LPD* laparoscopic pancreatoduodenectomy, *RPD* robot-assisted pancreatoduodenectomy, *TTFR* time to functional recovery, *LOHS* length of hospital stay, *CD* Clavien–Dindo classification, *POPF* postoperative pancreatic fistula, *NA* not available, ↓ indicates reduction, ↑ indicates increase, = indicates comparable outcome

#### PLOT

The PLOT trial was a single-center, non-blinded RCT conducted in India.^20^ Of 268 screened patients, 64 with peri-ampullary tumors were randomized in a 1:1 ratio to LPD or OPD.^20^ At the interim analysis, the primary outcome variable was changed from complication rate to length of hospital stay, in order to achieve an adequate sample size. LPD required longer operative time but reduced intraoperative blood loss (mean 401 mL vs. 250 mL; *p* < 0.001), and shorter hospital stay (13 vs. 7 days; *p* = 0.001) compared with OPD. Other short-term surgical and oncologic outcomes, including major morbidity, mortality, rates of postoperative pancreatic fistula, delayed gastric emptying, postoperative hemorrhage, lymph node yield, and R0 resection, were all comparable between both groups.

#### PADULAP

The second RCT on LPD versus OPD was a single-center, non-blinded, RCT from Spain.^21^ In total, 86 patients were screened and 66 adult patients with benign, premalignant, or malignant pancreatic tumors were included. The primary outcome of this trial was also length of hospital stay. This trial also confirmed the advantage of LPD in terms of shorter hospital stay (13.5 vs. 17 days; *p* = 0.024).^21^ Furthermore, longer median operative time (486 vs. 365 min; *p* < 0.001) and fewer Clavien–Dindo grade III or higher complications (5 vs. 11 patients; *p* = 0.04) were reported. Oncological outcomes (lymph node yield and R0 margin) and pancreas-specific complications were comparable between groups.

#### LEOPARD-2

The LEOPARD-2 trial was a multicenter, patient-blinded, randomized controlled phase II/III trial comparing laparoscopic and open pancreatoduodenectomy.^22^ Four centers participated; all performed 20 or more pancreatoduodenectomies annually and 20 LPDs before trial participation. Patients with a benign, premalignant, or malignant tumor without vascular involvement could be included. The trial was separated into a phase II and phase III trial; all patients randomized in phase II were included in phase III. The primary outcome of phase II was safety (complications and mortality), and the primary outcome of phase III was time to functional recovery (as previously defined for the LEOPARD trial). Between May 2016 and November 2017, 105 patients were randomized (of the projected sample size of 136 patients), of whom 99 underwent surgery—50 in the LPD group and 49 in the OPD group. The trial was prematurely terminated by the Data and Safety Monitoring Board because of a difference in 90-day complication-related mortality: 5 (10%) of 50 patients in the LPD group versus 1 (2%) of 49 patients in the OPD group (risk ratio [RR] 4.90, 95% CI 0.59–40.44; *p* = 0.20). The median time to functional recovery was 10 days (95% CI 5–15) after LPD versus 8 days (95% CI 7–9) after OPD (log-rank *p* = 0.80). Clavien–Dindo grade III or higher complications and grade B/C postoperative pancreatic fistula were comparable between the groups.

#### Ongoing Trials

The search identified three ongoing trials on MIPD.

First, a large, multicenter, prospective, randomized controlled, parallel-group, superiority trial (the TJDBPS01 trial; trial registry: NCT03138213) has been set-up in 14 centers in China.^23^ Centers participating have performed more than 104 LPDs each. A total of 656 adult patients with pancreatic or peri-ampullary malignancy are randomly allocated to LPD or OPD in a 1:1 ratio. The trial hypothesis is that LPD has superior or equivalent safety and advantages in postoperative recovery compared with OPD. The primary outcome is postoperative length of hospital stay. The enrolment period is scheduled to end in 2020 and the trial is awaited to be completed in 2022. When completed, this will be the largest RCT on MIPD to date.

Second, the PORTAL trial, also from China (trial registry: NCT04400357), is a multicenter, phase III, patient-blinded, non-inferiority trial that aims to primarily assess and compare the time to functional recovery of patients who undergo robot-assisted versus open pancreatoduodenectomy for benign and malignant lesions of the head of the pancreas. Secondary outcome measures will be overall complication rates, mortality, oncological outcomes, costs, and quality of life. The calculated sample size is 244 patients and follow-up will be completed up to 2 years after surgery. The results of this trial are expected to be published in 2024.

The third ongoing trial from Johns Hopkins (trial registry: NCT04171440) is a single-blinded, randomized trial comparing MIPD (robot-assisted and laparoscopy) with OPD. All patients with a benign, premalignant, or resectable malignant tumor are eligible for inclusion. The primary outcome of this trial is time to functional recovery, and the projected sample size is 240 patients in total. This trial started in February 2020 and is awaited to be completed in 2024.

## Discussion

Several RCTs comparing minimally invasive with open pancreatic resections have been published in recent years or are ongoing. For MIDP, the outcomes of two completed RCTs (LEOPARD and LAPOP) clearly show an advantage for MIDP in terms of functional recovery, hospital stay, and blood loss. Costs were at least comparable between both procedures. For tumors confined to the pancreas and procedures performed in centers with sufficient experience, MIDP seems to be the technique of choice. The role of MIPD remains a subject to debate since the outcomes of the three published RCTs (PLOT, PADULAP, LEOPARD-2) are conflicting.^20^–^22^ Although two single-center trials showed outcomes in favor of LPD in terms of hospital stay and complications, the only multicenter trial was terminated prematurely due to concerns regarding safety (higher mortality in the LPD group).

Currently, four trials on MIDP are recruiting (Table 1). The first trial, the DISPACT-2 trial, is including all types of tumors and comparing MIDP with ODP in regard to complications. Although the two recently completed RCTs did not show a difference in major morbidity after MIDP and ODP, both trials were not powered to show such a difference. Therefore, the DISPACT-2 trial will add additional information to the current body of evidence. The other three recruiting trials are focusing on oncological outcomes after MIDP compared with ODP. This illustrates the lack of evidence regarding the oncological safety of MIDP, which was already highlighted in an international survey where 21% of pancreatic surgeons reported viewing PDAC as a contraindication for MIDP.^24^,^25^ A systematic review including all available literature on MIDP versus ODP for PDAC showed that patients undergoing MIDP had smaller tumors with less perineural and lymphovascular invasion, indicating treatment allocation bias.^26^ Survival, R0 resection rate, and use of adjuvant chemotherapy were comparable, but a lower lymph node yield was seen after MIDP. The lower lymph node yield and signs of treatment allocation bias showed that the oncological safety of MIDP remains uncertain. The three upcoming trials, all comparing LDP with ODP for oncological outcomes (R0 resection rate, 2-year survival, and 2-year disease-free survival) in a multicenter setting, with sample sizes of over 200 patients, are expected to provide the relevant answers.

The three trials on MIPD had conflicting results. Several differences between the three completed trials are present. First, the PLOT and PADULAP trials were single-center trials, whereas the LEOPARD-2 trial included patients from four centers. In addition, a difference in experience was present. The center that performed the PLOT trial performed over 150 LPDs before the start of the trial, and the surgeon performing the PADULAP trial had performed 25 LPDs before the start of the trial but had performed over 250 laparoscopic gastric bypass procedures. In the LEOPARD-2 trial, participating surgeons had performed 23–24 LPDs before the start of the trial. These differences in experience could, to some extent, explain the differences in outcome. From the available literature comparing LPD with OPD, it is thought that in experienced hands, in high-volume centers (at least 20 LPDs/year), LPD has the advantage of shorter hospital stay, less blood loss, and potential improved quality of life, with comparable morbidity and mortality.^27^–^29^ Therefore, an annual volume of a minimum of 20 LPDs (and robot-assisted procedures) is recommend by the Miami guidelines on MIPRs.^30^ Annual volume could also be a cause of the differences in outcome of the trials. Centers participating in the LEOPARD-2 trial were performing 20 LPDs annually before the start of the trial. Due to randomization, these annual numbers were halved, to a median of 11 procedures. This reduced volume during the trial could have influenced the outcomes of the LEOPARD-2 trial in a negative way.

Upcoming trials comparing MIPD with OPD are all large trials from experienced centers. One trial will include only laparoscopic procedures, whereas another trial will compare both laparoscopic and robot-assisted procedures with the open procedure. The third trial will be the first trial to compare robot-assisted pancreatoduodenectomy and OPD. The outcomes of these trials will provide strong evidence on the possible benefits of MIPD compared with OPD in a high-volume setting.

## Conclusion

The number of completed and recruiting trials on MIPR shows the need for more strong evidence regarding the advantages of MIDP and MIPD. Current literature is promising but does not provide the answers to all questions. MIDP can be considered as the preferred approach for benign and low-grade malignant tumors in selected patients. Oncological safety of MIDP in the treatment of malignancies should be further evaluated. Though it has been shown that MIPD is not inferior to the open approach in safety and feasibility, the overall benefits still need to be verified. Completion and publication of the trials currently recruiting are awaited with great interest.
